# Gastric involvement in multiple myeloma-associated AL amyloidosis: a case with serial endoscopy and a pooled analysis of 30 published cases

**DOI:** 10.3389/fonc.2026.1960604

**Published:** 2026-10-06

**Authors:** Li Wang, Jiateng You, Gong Cheng, Xiaohui Shen

**Affiliations:** 1 Department of Hematology, Gansu Provincial Hospital of TCM, Lanzhou, China; 2 The Second Clinical Medical College, Lanzhou University, Lanzhou, China; 3 Department of Gastroenterology, Gansu Provincial Hospital, Lanzhou, China; 4 The Third Clinical Medical College, Lanzhou University, Lanzhou, China

**Keywords:** AL amyloidosis, gastric amyloidosis, gastric outlet obstruction, gastrointestinal bleeding, light-chain amyloidosis (AL), multiple myeloma

## Abstract

**Objective:**

To describe serial endoscopic findings in a patient with relapsed/refractory multiple myeloma (MM) and gastric amyloidosis and to summarize 30 published cases, with emphasis on their clinical, endoscopic, and histopathologic features and common diagnostic challenges.

**Methods:**

This study comprised a case report and an individual-patient pooled analysis of published cases. We reviewed the clinical course and serial endoscopic assessments of the index patient and searched PubMed, Web of Science, Cochrane Library, and Embase from inception through June 1, 2026. Reports of MM-associated gastric amyloidosis with extractable clinical information were included. Demographic characteristics, symptoms, sites of involvement, endoscopic findings, diagnostic methods, clonal characteristics, treatment, and outcomes were analyzed descriptively.

**Results:**

During myeloma relapse, the index patient developed refractory nausea and vomiting. Routine histopathologic examination of a biopsy from a proliferative lesion in the gastric fundus showed a fundic gland polyp, while Congo red staining was weakly positive. Symptoms improved substantially after anti-multiple myeloma treatment was resumed, and repeat endoscopy after two cycles of anti-multiple myeloma treatment showed fewer and smaller polypoid elevations. As the disease subsequently progressed, nausea and vomiting recurred intermittently and the polypoid elevations gradually increased, raising the possibility that the gastric manifestations tracked plasma-cell clonal activity. Thirty previously published patients were included. Bleeding-related manifestations were most common (20/30, 66.7%), followed by nausea and vomiting (10/30, 33.3%). The antrum was the most frequently involved site (16/26, 61.5%). Endoscopic findings were available for 28 patients; nodules, polyps, masses, or other nonhematomatous elevated lesions predominated (16/28, 57.1%). Among 25 patients with a clear diagnostic sequence, 14/25 (56.0%) were diagnosed with MM or smoldering multiple myeloma after gastrointestinal amyloid deposition was identified, whereas 11/25 (44.0%) developed gastric involvement in the setting of previously diagnosed MM.

**Conclusions:**

MM-associated gastric amyloidosis lacks specific symptoms and endoscopic features. Bleeding, refractory vomiting or obstruction, and tumor-like lesions are important presentations. In patients with MM who have unexplained persistent gastrointestinal symptoms, or discordance among the clinical presentation, endoscopic phenotype, and histopathologic findings, multiple adequate biopsies should be obtained, with deeper sampling when necessary, followed by Congo red staining, confirmation under polarized light, and amyloid protein typing.

## 1 Introduction

Multiple myeloma (MM) is a malignant plasma-cell disorder characterized by clonal proliferation. Monoclonal immunoglobulins or free light chains produced by abnormal plasma cells are central to diagnosis and response assessment, but they may also misfold, form amyloid fibrils, and deposit in multiple organs. Previous studies have estimated that approximately 10%–15% of patients with MM develop AL amyloidosis involving vital organs (1). Although this overlap is not uncommon, clinical recognition remains focused largely on typical target organs such as the heart and kidneys. Renal, cardiac, hepatic, peripheral nerve, and soft-tissue involvement is more readily recognized, whereas gastrointestinal involvement, particularly gastric involvement, often lacks specific manifestations and may be attributed to ordinary gastritis, peptic ulcer disease, adverse drug effects, gastric cancer, mucosa-associated lymphoid tissue (MALT) lymphoma, or gastric dysmotility. Sidiqi and Gertz (2) noted that patients with monoclonal gammopathy of undetermined significance (MGUS), smoldering multiple myeloma (SMM), or active MM who develop weight loss, early satiety, edema, exertional dyspnea, diarrhea, or other multisystem manifestations unexplained by common disorders should undergo evaluation for amyloidosis rather than having these findings attributed solely to progression of the underlying disease or treatment-related effects. Ebert and Nagar (3) further described amyloid deposition in the mucosa, submucosa, vascular walls, and neuromuscular structures of the gastrointestinal tract, with clinical manifestations including bleeding, diarrhea, constipation, malabsorption, and impaired gastric emptying. MM-associated gastric amyloidosis therefore represents the convergence of a plasma-cell clonal disorder and light-chain-mediated organ injury rather than a purely local gastric disease.

Recognition of gastric amyloidosis is complicated by a discrepancy between infrequent clinically overt involvement and potentially widespread histologic deposition. Menke et al. (4) reviewed 769 patients with primary systemic amyloidosis. Gastrointestinal biopsy confirmed amyloid deposition in 59/769 (7.7%), whereas symptomatic gastric amyloidosis occurred in only 8/769 (1.0%). The latter group mainly presented with hematemesis or chronic nausea and vomiting accompanied by weight loss and had poor outcomes. Yamada et al. (5) performed autopsies in 21 patients with systemic amyloidosis, 18 of whom had AL amyloidosis. Gastrointestinal vascular amyloid deposition was present in 18/18 (100.0%), and deposition in the lamina propria and muscularis mucosae of the stomach was more frequent and pronounced than that in the rectum. These data show that clinical symptoms, conventional endoscopic findings, and tissue deposition do not necessarily parallel one another. Deposition may be widespread but become clinically overt only when specific tissue layers are involved and bleeding, obstruction, or dysmotility develops. Diagnosis therefore depends not only on the presence of amyloid but also on the site and depth of biopsy, whether Congo red staining is specifically requested, and whether amyloid protein typing can be completed.

Published cases show that MM-associated gastric amyloidosis may present with upper gastrointestinal bleeding, recurrent melena, refractory nausea and vomiting, gastric retention, gastric outlet obstruction, refractory ulcers, tumor-like or polypoid lesions, and submucosal hematoma. Gjeorgjievski et al. (6) described a patient with SMM who presented with melena and upper gastrointestinal bleeding. Endoscopy revealed a large blood clot and an ulcerated mass, and gastric amyloidosis was established by Congo red staining and mass spectrometry. The patient reported by Singh et al. (7) had MM and presented primarily with hematemesis, melena, and multiple gastric ulcers. Shih et al. (8) described a patient who initially had an antral ulcer refractory to acid-suppressive therapy; the combination of proteinuria, cardiac involvement, and bone marrow findings ultimately established MM-associated light-chain amyloidosis. These cases demonstrate that endoscopic morphology alone cannot confirm or exclude gastric amyloidosis. Diagnosis requires integration of the underlying plasma-cell disorder, persistence of symptoms, response to conventional therapy, histopathologic morphology, and special staining results.

We report a patient with IgAλ MM who achieved a very good partial response (VGPR) twice, discontinued treatment on both occasions, and subsequently relapsed. Recurrent refractory nausea and vomiting developed during relapse. Endoscopy showed proliferative lesions in the gastric fundus and body. Routine histopathology identified only fundic gland polyps, while Congo red staining performed at an external laboratory was weakly positive. After anti-multiple myeloma treatment was resumed, the nausea and vomiting improved substantially; repeat endoscopy after two cycles of anti-multiple myeloma treatment showed fewer and smaller polypoid elevations. With subsequent disease progression, nausea and vomiting again occurred intermittently, and serial endoscopy showed a gradual increase in the polypoid elevations. This temporal pattern raises the possibility that the gastric symptoms and endoscopic lesions were associated with plasma-cell clonal activity and light-chain-related tissue deposition. We also searched PubMed, Web of Science, Cochrane Library, and Embase and identified 30 previously published patients. Their demographic characteristics, symptoms, sites of gastric involvement, endoscopic phenotypes, diagnostic methods, immunoglobulin types, light-chain types, diagnostic sequence, imaging findings, autopsy and deep-biopsy findings, treatment responses, and outcomes were summarized to inform more precise diagnosis and management in gastroenterology and hematology practice. To our knowledge, this case provides the first serial endoscopic documentation of dynamic changes in gastric lesions in relation to the response to anti-multiple myeloma treatment and subsequent disease progression, offering longitudinal endoscopic evidence of a potential association between gastric manifestations and plasma-cell clonal activity. Drawing on the index case and 30 published cases, we further propose the first clinical-endoscopic framework comprising five phenotypes of gastric amyloidosis: bleeding, obstructive, tumor-like, ulcerative, and occult. This framework may facilitate clinical recognition and stratified assessment of the disease.

## 2 Case presentation

A 74-year-old woman presented to another hospital with low back pain in December 2018. Lumbar computed tomography (CT) showed a vertebral compression fracture, mild lumbar spondylolisthesis, and degenerative changes in the lumbar intervertebral discs. As the pain progressively worsened, she was referred to our hospital and admitted with a preliminary diagnosis of lumbar disc herniation. Repeat thoracolumbar CT after admission showed multiple vertebral abnormalities and compression fractures of T12, L1, and L5; the radiology report recommended exclusion of myeloma or metastatic disease. Complete blood count showed a white blood cell count of 3.31 × 10^9^/L, neutrophil count of 2.14 × 10^9^/L, hemoglobin level of 89 g/L, and platelet count of 150 × 10^9^/L. Biochemical testing showed total protein 81.49 g/L, globulin 69.9 g/L, creatinine 81 μmol/L, calcium 2.08 mmol/L, lactate dehydrogenase 111 U/L, and β_2_-microglobulin 2.2 mg/L (detailed laboratory results are shown in **Figure 1**). MM was suspected because of the multiple vertebral abnormalities and compression fractures accompanied by anemia and marked hyperglobulinemia. Serum and urine protein electrophoresis, immunofixation electrophoresis, serum free light-chain testing, bone marrow aspiration and biopsy, flow cytometry, and fluorescence *in situ* hybridization (FISH) were subsequently performed. Serum M-protein was 36.4 g/L, serum free λ light chain was 528.33 mg/L, and the κ/λ ratio was 0.02. Plasma cells accounted for 16% of bone marrow cells, malignant monoclonal plasma cells accounted for 4.5% by flow cytometry, FISH showed 1q21 amplification and t(4;14), and the karyotype was normal. These findings established a diagnosis of IgAλ MM, Durie–Salmon stage IIIB, International Staging System (ISS) stage III, and Revised International Staging System (R-ISS) stage III. Anti-multiple myeloma treatment was initiated with the PAD regimen: bortezomib 2.3 mg subcutaneously on days 1, 4, 8, and 11; pirarubicin 10 mg intravenously on days 1–4; and dexamethasone 20 mg intravenously on days 1, 2, 4, 5, 8, 9, 11, and 12. Eight cycles were completed. In November 2020, the response was assessed as VGPR. Because her low back pain and other symptoms had improved substantially, the patient discontinued treatment on her own.

**Figure 1:**
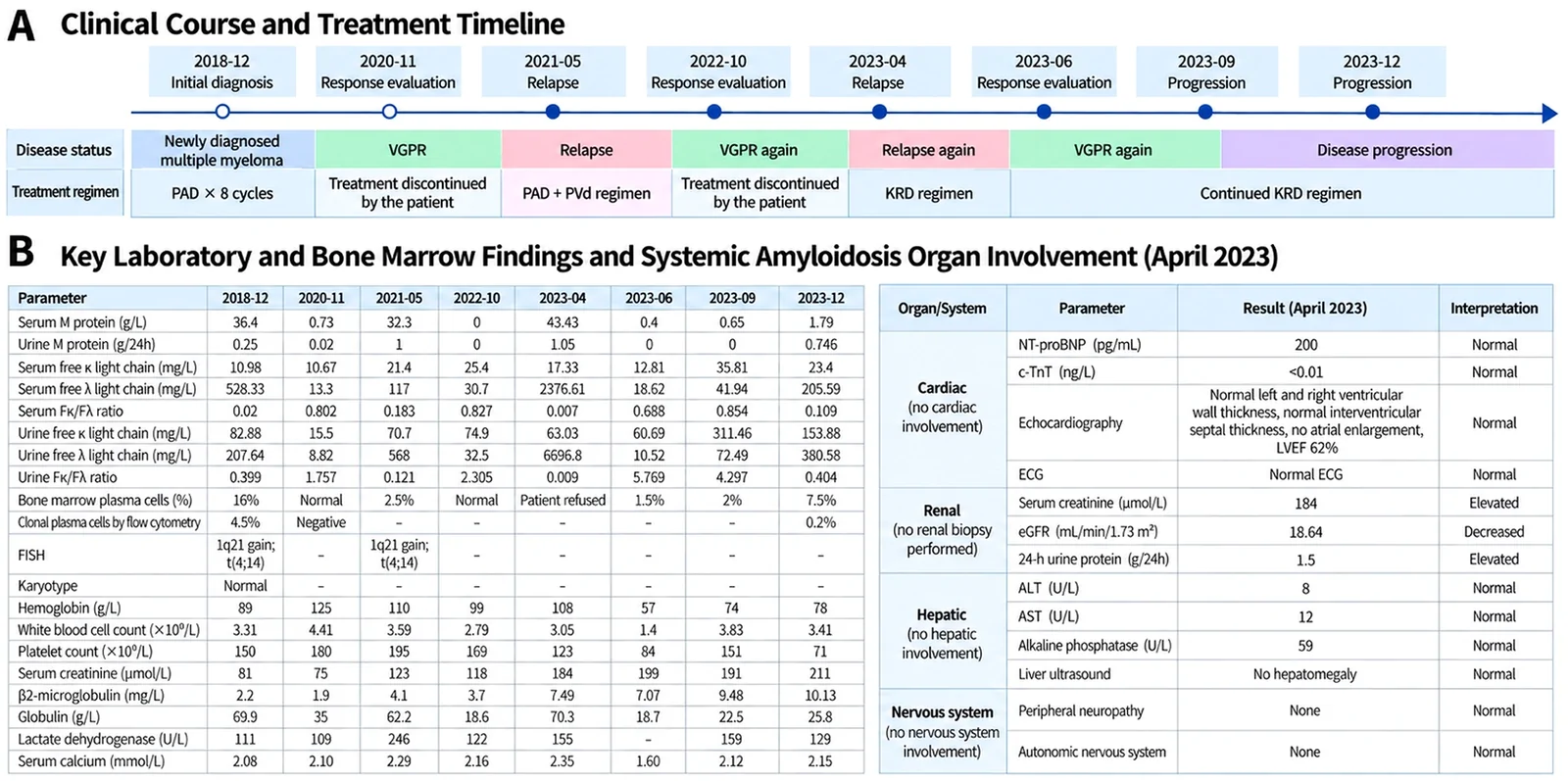
**(A)** Clinical course and treatment timeline. **(B)** Key Laboratory and Bone Marrow Findings and Systemic Amyloidosis Organ Involvement (April 2023).

In May 2021, the patient was admitted to the respiratory medicine department of our hospital with pleural effusion. She underwent closed thoracic drainage and antimicrobial therapy before transfer to the hematology department. Further evaluation showed relapsed MM. Treatment was resumed with the PAd regimen and subsequently the PVd regimen; the latter comprised pomalidomide 4 mg orally on days 1–21, bortezomib 2.3 mg subcutaneously on days 1, 4, 8, and 11, and dexamethasone 20 mg intravenously on days 1, 2, 4, 5, 8, 9, 11, and 12. In October 2022, she again achieved VGPR. Ixazomib maintenance therapy was recommended, but she declined and again discontinued treatment. In April 2023, she developed recurrent refractory nausea and vomiting, accompanied by chest tightness and dyspnea. The vomitus consisted of gastric contents, and continuous oxygen therapy was required. Bone marrow aspiration, bone marrow biopsy, and other examinations were performed to reassess the disease comprehensively and showed another relapse. Recurrent nausea and vomiting were the principal symptoms during this admission, and the initial evaluation and management focused on these manifestations.

Upper gastrointestinal endoscopy showed proliferative lesions in the gastric fundus and body of uncertain nature; a MALT-related lesion was considered endoscopically (**Figure 2A**). Routine histopathologic examination of a fundic biopsy showed a fundic gland polyp, Helicobacter pylori testing was negative, and Congo red staining performed at an external laboratory was weakly positive (**Figures 3A, B**). The patient’s established IgAλ MM, refractory upper gastrointestinal symptoms during relapse, discordance between symptom severity and routine histopathologic findings, and weakly positive Congo red staining supported clinical consideration of gastric amyloidosis. For symptomatic treatment, the patient initially received the 5-hydroxytryptamine type 3 receptor antagonist ondansetron without meaningful relief. The neurokinin-1 receptor antagonist aprepitant and the glucocorticoid dexamethasone were subsequently added, reducing the frequency of nausea, although nausea and vomiting persisted. On April 21, 2023, treatment with the KRd regimen was initiated: carfilzomib 30 mg intravenously on days 1 and 2 of cycle 1, followed by 43 mg intravenously on days 1, 2, 8, 9, 15, and 16; lenalidomide 25 mg orally on days 1–21; and dexamethasone 40 mg intravenously on days 1, 8, 15, and 22. Nausea and vomiting gradually improved. In June 2023, after two cycles of treatment, these symptoms had improved substantially, and repeat endoscopy showed fewer and smaller polypoid elevations (**Figure 2B**). Although anti-multiple myeloma treatment was continued as scheduled, the disease progressed and nausea and vomiting recurred intermittently. Repeat endoscopy in September 2023 (**Figure 2C**) and December 2023 (**Figure 2D**) showed a gradual increase in the polypoid elevations. The patient and her family subsequently declined further active treatment, and she died in April 2024.

**Figure 2:**
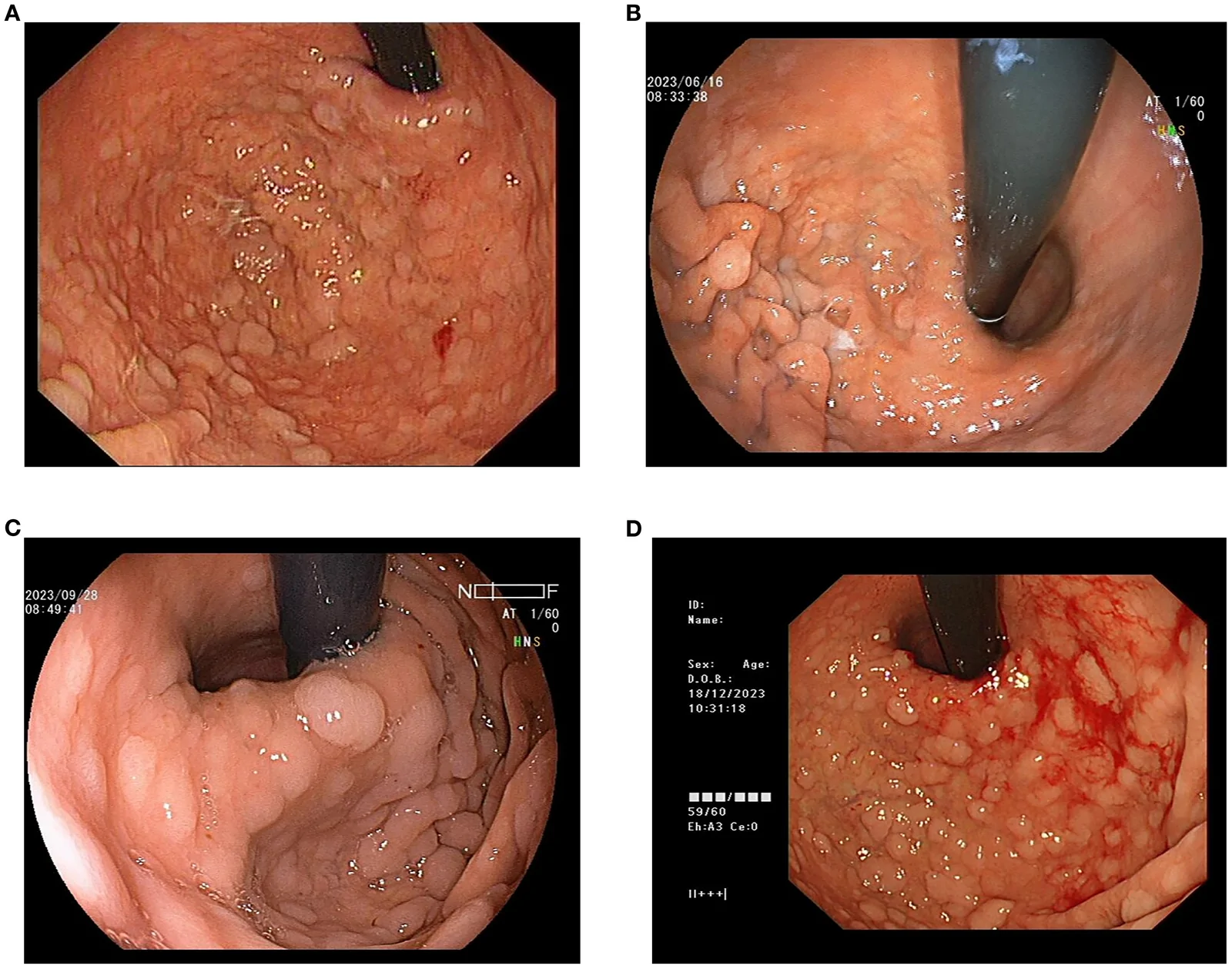
**(A)** Gastroscopy images from April 2023. **(B)** Gastroscopy image after anti-multiple myeloma treatment on June 16, 2023. **(C)** Gastroscopy image from September 28, 2023. **(D)** Gastroscopy image from December 18, 2023.

**Figure 3:**
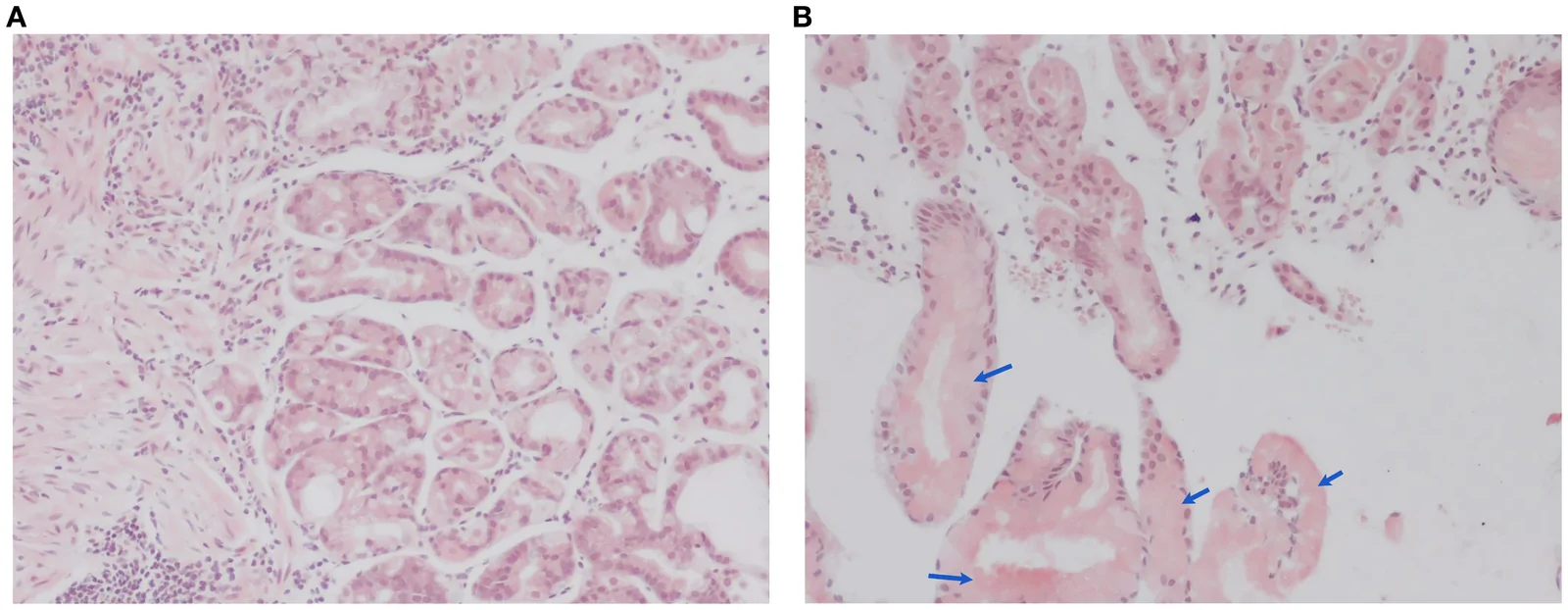
**(A)** Positive result of Congo red staining in histopathology ×40. **(B)** Positive result of Congo red staining in histopathology ×100.

Four aspects of this case warrant attention. First, refractory gastrointestinal symptoms coincided temporally with myeloma relapse. Second, endoscopy showed only nonspecific elevations that could readily be interpreted as polyps or lymphoid hyperplasia, whereas the severity of symptoms was disproportionate to the routine histopathologic findings. Third, conventional antiemetic therapy had limited efficacy, while symptoms improved after anti-multiple myeloma treatment was resumed, raising the possibility that the gastric manifestations were dynamically associated with plasma-cell clonal activity and light-chain-related tissue deposition. Fourth, after two cycles of treatment, nausea and vomiting improved substantially and repeat endoscopy showed fewer and smaller polypoid elevations; as the disease continued to progress, the elevations subsequently increased. This course supports the view that the gastric lesions were not simply static polypoid changes and may have varied with systemic disease activity.

## 3 Results of the pooled analysis

To define the clinical features of MM-associated gastric amyloidosis and systematically summarize the clinical presentations, endoscopic features, and diagnostic and therapeutic characteristics of published cases, we searched PubMed, Web of Science, Cochrane Library, and Embase. The search strategy encompassed three concepts: the underlying disease, amyloid deposition, and gastric involvement. Terms for the underlying disease included multiple myeloma, myeloma, and plasma cell myeloma; terms for amyloidosis included amyloidosis, amyloid deposition, and amyloid protein deposition; and terms for gastric involvement included gastric, stomach, gastric amyloidosis, stomach amyloidosis, and gastrointestinal amyloidosis. Reference lists of included reports were also screened to identify early case reports, non-English reports, and relevant publications potentially missed by the database searches. Reports were eligible if they met the following criteria: (1) MM or SMM; (2) histologic evidence of gastric amyloid deposition or an explicit diagnosis of gastric amyloidosis in the case report; and (3) extractable information on at least one of the following: symptoms, endoscopic findings, diagnosis, treatment, or prognosis. Reports of amyloidosis unrelated to a plasma-cell disorder, reports without gastric involvement, duplicate reports, and reports lacking essential case information were excluded. All databases were searched from inception through June 1, 2026. The search identified 1,026 records. After removal of 304 duplicates, 722 records underwent title and abstract screening, of which 674 were excluded for failing to meet the prespecified eligibility criteria. Full texts of 36 reports were then assessed. Six were excluded because individual-patient data could not be extracted (n = 1), essential case information was severely incomplete (n = 4), or the disease and histopathologic eligibility criteria were not met (n = 1). The final individual-patient pooled analysis included 30 publications reporting 30 patients (**Figure 4**).

**Figure 4:**
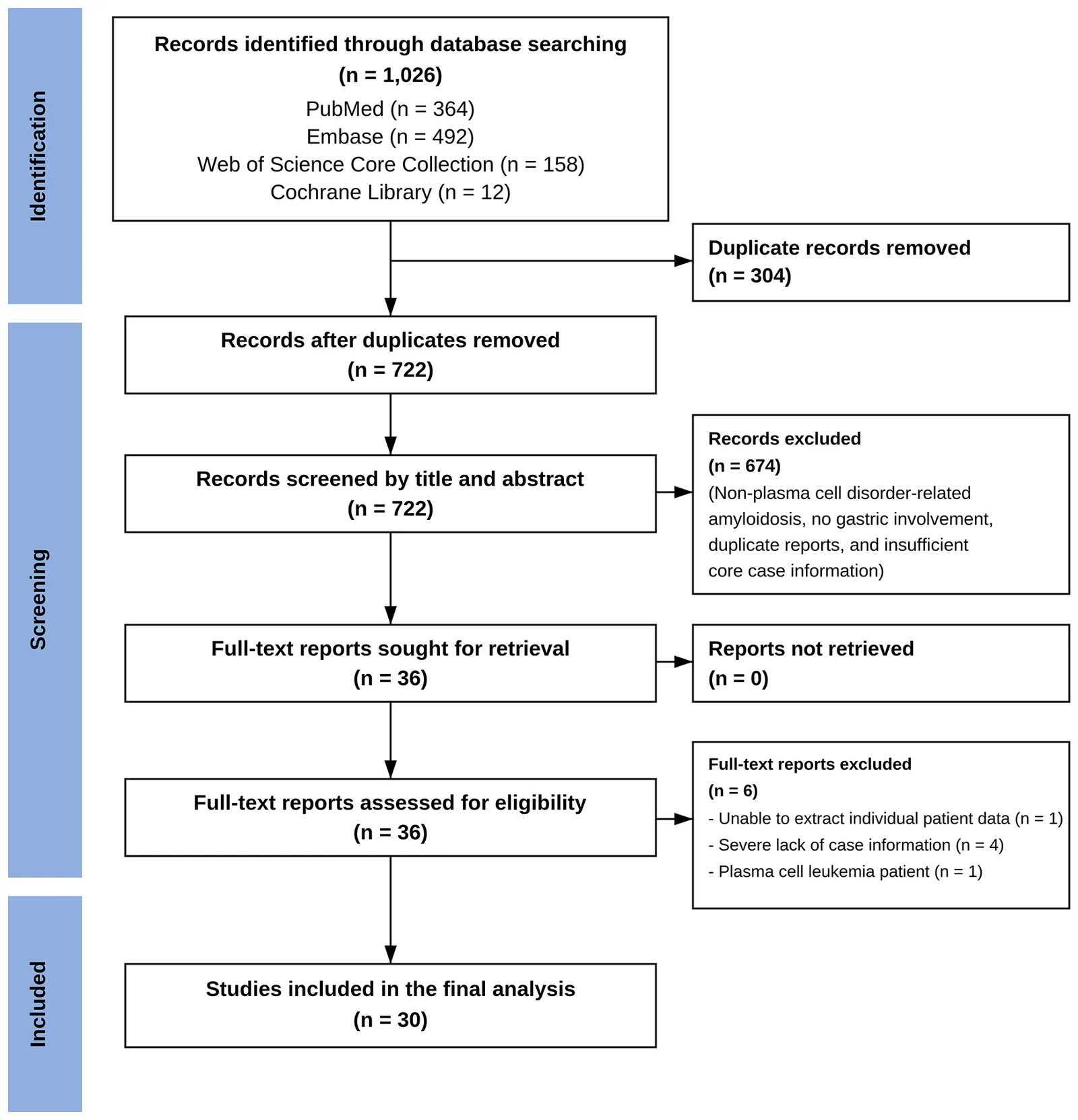
Flow diagram of literature screening for the pooled patient analysis.

Among the 30 included patients, 15/30 (50.0%) were male and 15/30 (50.0%) were female. Age ranged from 39 to 92 years, with a mean of 62.8 years and a median of 63 years. The underlying plasma-cell disorder was active MM in most patients, including the patient reported by Cryer and Kissane (9); the remaining cases included SMM (6, 10). The main clinical characteristics are summarized in **Table 1**. For each variable, the denominator was the number of patients with clearly documented data. Symptoms, sites of gastric involvement, and endoscopic findings could overlap within the same patient. Extracted information for each case is provided in **Supplementary Table 1**.

**Table 1:** Clinical characteristics of the 30 patients included in the literature-based pooled analysis.

Variable	Result
Included cases	Initially identified, n = 36; excluded, n = 6; included, n = 30
Sex (N = 30)	
Male	15/30 (50.0%)
Female	15/30 (50.0%)
Age (N = 30)	39–92 years; mean, 62.8 years; median, 63 years
Clinical symptoms and bleeding-related manifestations (N = 30)	
Bleeding-related manifestations (symptoms or endoscopic evidence)	20/30 (66.7%)
Hematemesis (subtypes may overlap)	9/30 (30.0%)
Hematochezia (subtypes may overlap)	3/30 (10.0%)
Melena (subtypes may overlap)	4/30 (13.3%)
Nausea and vomiting	10/30 (33.3%)
Abdominal pain, bloating, dyspepsia, poor appetite, or anorexia	10/30 (33.3%)
Gastric retention, pyloric stenosis, gastric outlet obstruction, or obstructive manifestations	7/30 (23.3%)
Sites of gastric involvement (N = 26; categories may overlap)	
Antral involvement	16/26 (61.5%)
Gastric body involvement	7/26 (26.9%)
Pyloric involvement	6/26 (23.1%)
Fundic involvement	3/26 (11.5%)
Gastric angle involvement	2/26 (7.7%)
Endoscopic findings (N = 28; categories may overlap)	
Nodules, polyps, masses, or other nonhematomatous elevated lesions	16/28 (57.1%)
Ulcers or erosions	9/28 (32.1%)
Bleeding, oozing, contact bleeding, or blood clots	9/28 (32.1%)
Submucosal hematoma	4/28 (14.3%)
Stenosis, obstruction, or gastric retention	6/28 (21.4%)
Hyperemia, edema, erythema, gastritis-like changes, coarse mucosa, or gastric wall rigidity	9/28 (32.1%)
Normal or only minor abnormalities	2/28 (7.1%)
Diagnostic method (N = 28)	
Histologic diagnosis; Congo red staining not explicitly reported	11/28 (39.3%)
Tissue sampling with Congo red staining	17/28 (60.7%)
Diagnostic sequence (N = 25)	
Gastric or gastrointestinal amyloidosis before MM or SMM	14/25 (56.0%)
MM before gastric amyloidosis	11/25 (44.0%)
Immunoglobulin heavy-chain type (N = 6): IgG, 6/6 (100.0%)	
Light-chain type (N = 12)	
κ light chain	8/12 (66.7%)
λ light chain	4/12 (33.3%)
Treatment outcome (N = 15)	
Death	8/15 (53.3%)
Improvement or short-term stability	6/15 (40.0%)
Transition to palliative care because anti-multiple myeloma treatment was not tolerated	1/15 (6.7%)

For each variable, N denotes the number of patients with clearly documented data. Symptoms, sites of gastric involvement, and endoscopic findings were non-mutually exclusive; a patient could be counted in more than one category, and the category percentages therefore should not be summed. Bleeding-related manifestations included overt bleeding symptoms, active endoscopic bleeding or oozing, blood clots, and submucosal hematoma. Symptom data were available for all 30 patients, whereas clear endoscopic findings were documented for 28; denominators were therefore 30 for symptoms and 28 for endoscopic findings.

Bleeding-related manifestations were the most common and were present in 20/30 patients (66.7%); 17/30 (56.7%) had overt bleeding manifestations. Hematemesis, melena, and hematochezia were reported in 9/30 (30.0%), 4/30 (13.3%), and 3/30 (10.0%), respectively, with overlap among subtypes (6, 7, 11–17). Nausea and vomiting occurred in 10/30 (33.3%); abdominal pain, bloating, dyspepsia, poor appetite, or anorexia in 10/30 (33.3%); and gastric retention, pyloric stenosis, gastric outlet obstruction, or obstructive manifestations in 7/30 (23.3%) (9, 18–22).

The site of gastric involvement was clearly reported in 26 patients. The antrum was most frequently involved (16/26, 61.5%), followed by the gastric body (7/26, 26.9%), pylorus (6/26, 23.1%), fundus (3/26, 11.5%), and gastric angle (2/26, 7.7%). Multiple gastric regions could be involved in the same patient, and some patients also had duodenal, colonic, or esophageal involvement (9, 10, 18, 20, 23).

Endoscopic findings were clearly reported in 28 patients. Nodules, polyps, masses, or other nonhematomatous elevated lesions were most common (16/28, 57.1%). Ulcers or erosions were present in 9/28 (32.1%); overt bleeding, oozing, contact bleeding, or blood clots in 9/28 (32.1%); submucosal hematoma in 4/28 (14.3%); stenosis, obstruction, or gastric retention in 6/28 (21.4%); and hyperemia, edema, erythema, gastritis-like changes, coarse mucosa, or gastric wall rigidity in 9/28 (32.1%). Endoscopy was nearly normal or showed only minor abnormalities in 2/28 patients (7.1%). These findings were not mutually exclusive (24–32).

Diagnostic methods were described in detail for 28 patients, all of whom were diagnosed histologically from gastrointestinal biopsy or surgical specimens (28/28, 100.0%). Congo red staining was explicitly reported in 17/28 (60.7%); the remaining 11/28 (39.3%) were reported as having a routine histologic diagnosis without specifying whether Congo red staining had been performed. Some reports also used polarized light microscopy, immunohistochemical demonstration of light-chain restriction, or mass spectrometric typing. Gjeorgjievski et al. (6) performed liquid chromatography-tandem mass spectrometry on the Congo red-positive area and identified ALλ amyloid.

Immunoglobulin heavy-chain type was reported in six patients, all of whom had IgG (6/6, 100.0%) (6, 16, 18, 25, 31, 33). Light-chain type was reported in 12 patients: κ in 8/12 (66.7%) and λ in 4/12 (33.3%) (6, 8, 16, 17, 23–25, 31, 33–36). Heavy-chain type was not reported in the other 24 patients, and light-chain type was not reported in the other 18, underscoring the incomplete clonal characterization in published case reports. The index patient had IgAλ MM.

The diagnostic sequence was clear in 25 patients. In 14/25 (56.0%), tissue examination prompted by gastric or gastrointestinal symptoms identified amyloid deposition, followed by a diagnosis of MM or SMM. In 11/25 (44.0%), gastric involvement was identified in the setting of previously diagnosed MM. Together, these two pathways accounted for 25/25 patients (100.0%). In the index case, MM was diagnosed before gastric involvement was recognized.

Specific imaging findings were reported in three patients: concentric thickening of the gastric antrum and pylorus, marked gastric dilatation with collapse of the proximal duodenum, and a heterogeneously dense, intensely enhancing mass-like lesion within the antrum (19, 25, 34). In addition, autopsy findings reported by Yamane et al. (37) showed extensive gastrointestinal involvement with mural thickening or stenosis; amyloid was deposited in the muscularis mucosae, submucosal vascular walls, and muscularis propria. Reithmeier et al. (31) documented involvement of the entire gastrointestinal tract by deep biopsy.

Outcomes were clearly reported in 15 patients. Death occurred in 8/15 (53.3%); improvement in symptoms, cessation of bleeding, regression of gastric lesions, or short-term postoperative stability was reported in 6/15 (40.0%); and 1/15 (6.7%) transitioned to palliative care because anti-multiple myeloma treatment was not tolerated. Outcomes were not clearly reported for the remaining 15 patients (**Table 1**).

## 4 Discussion

The principal clinical value of this case lies not in the isolated finding of weakly positive Congo red staining in lesions of the gastric fundus and body, but in the longitudinal concordance among refractory nausea and vomiting, endoscopic changes, and myeloma disease activity. The patient achieved VGPR in 2020 and 2022, discontinued treatment on both occasions, and subsequently relapsed. During relapse in April 2023, recurrent refractory nausea and vomiting developed and responded only partially to conventional antiemetic therapy. Symptoms gradually improved after anti-multiple myeloma treatment was resumed. After two treatment cycles in June 2023, nausea and vomiting had improved substantially, while repeat endoscopy showed fewer and smaller polypoid elevations. Despite continued anti-multiple myeloma treatment, the disease progressed, nausea and vomiting recurred intermittently, and endoscopy in September and December 2023 showed a gradual increase in the polypoid elevations. The gastric symptoms and endoscopic lesions may therefore have been dynamically associated with plasma-cell clonal burden, light-chain-related tissue injury, and the extent of gastric wall deposition rather than merely coexisting by chance. The improvement in symptoms and lesions after systemic therapy and their subsequent worsening with disease progression are consistent with regression of a gastric amyloid mass after anti-multiple myeloma treatment reported by Bhagat et al. (23), improvement of gastric outlet obstruction after plasma-cell-directed therapy reported by Cohen et al. (19), and healing of a refractory gastric ulcer after systemic therapy reported by Shih et al. (8).

Immunoglobulin heavy-chain and light-chain types were a focus of this analysis. Previous studies of AL amyloidosis have concentrated on the amyloidogenic potential and variable-region structure of the light chain itself. Gastric involvement, however, has been reported in few and scattered cases, and systematic data on clonal type remain limited. Among the 30 patients in this pooled analysis, only six had a reported heavy-chain type; all six had IgG (6/6, 100.0%). Light-chain type was reported in 12 patients, including κ in 8/12 (66.7%) and λ in 4/12 (33.3%). These results primarily reflect incomplete clonal information in published case reports and do not establish a preferential heavy- or light-chain type in gastric amyloidosis. The IgAλ type in the index case also shows that gastric amyloidosis can occur in non-IgG myeloma. Tissue deposition and organ injury may depend more on the structural stability, propensity for misfolding, serum burden, tissue microenvironment, and deposition site of the abnormal light chain.

The diagnostic sequence delineated two clinical pathways. Among the 25 patients with a clear sequence, gastric or gastrointestinal amyloidosis was recognized first in 14/25 (56.0%), whereas MM was diagnosed first in 11/25 (44.0%). Patients in the former group commonly entered the gastroenterology pathway because of gastrointestinal bleeding, a gastric cancer-like lesion, a refractory ulcer, submucosal hematoma, gastric outlet obstruction, or otherwise unexplained gastrointestinal symptoms; subsequent histopathologic and hematologic evaluation then identified the plasma-cell disorder. The latter pathway emphasizes the need to assess new organ involvement during hematology follow-up. In patients with known myeloma, particularly during relapse, treatment interruption, rising light-chain levels, declining response, or multisystem manifestations, refractory nausea and vomiting, melena, hematemesis, gastric retention, gastric wall thickening, or endoscopic abnormalities should not be attributed automatically to treatment-related adverse effects, infection, ordinary gastritis, or peptic ulcer disease. The index patient discontinued treatment after achieving VGPR twice, relapsed on both occasions, and developed refractory nausea and vomiting during relapse. Her gastric symptoms may therefore have reflected disease activity and gastric involvement.

Mechanistically, gastric amyloidosis may manifest as bleeding, obstructive, tumor-like, ulcerative, or occult phenotypes (**Table 2**), largely according to the layer of amyloid deposition and the predominantly involved structure. Deposition in vascular walls may increase vascular fragility, cause local ischemia, and impair hemostasis, resulting in hematemesis, melena, oozing, large blood clots, or submucosal hematoma. The severe bleeding described by Khan et al. (14) in a patient with simultaneous gastric and transverse colonic involvement illustrates how vascular deposition, anticoagulant therapy, and coagulopathy may jointly increase bleeding risk. The submucosal hematomas reported by Kim et al. (24), Pak and Markovic (26), and Yanase et al. (25) further show that some patients develop an intramural hematoma over a short period rather than chronic low-volume oozing, thereby mimicking a submucosal tumor. Deposition in the lamina propria and submucosa more readily produces nodules, polyps, elevations, masses, ulcers, or gastric cancer-like changes. Involvement of the muscular layer, neural plexus, and pyloric region may cause gastric wall rigidity, impaired gastric emptying, gastric retention, and gastric outlet obstruction. This mechanism accounts for persistent vomiting and pyloric stenosis in the cases reported by Cryer and Kissane (9), Chapple et al. (18), Bhagat et al. (23), Lei et al. (20), and Yamane et al. (37). On this basis, we developed a layer-based clinicopathologic model of gastric amyloidosis that integrates the proposed mechanisms with the clinical manifestations (**Figure 5**).

**Table 2:** Clinical-endoscopic phenotypes of gastric amyloidosis and their potential mechanisms.

Phenotype	Representative clinical manifestations	Typical endoscopic or imaging findings	Potential pathologic basis	Common misdiagnoses	Clinical implications
Bleeding phenotype	Hematemesis, melena, hematochezia, or active or subacute bleeding; abdominal pain in some patients	Ulcers, blood clots, oozing, contact bleeding, submucosal hematoma, or an intensely enhancing mass	Mucosal and submucosal vascular deposition causing increased vascular fragility, local ischemia, or intramural bleeding	Peptic ulcer disease, drug-related bleeding, tumor bleeding, gastric submucosal tumor, gastrointestinal stromal tumor, or hemangioma	Unexplained bleeding or submucosal hematoma should prompt Congo red staining
Obstructive phenotype	Refractory nausea and vomiting, gastric retention, and feeding intolerance	Gastric dilatation, pyloric stenosis, duodenal collapse, and gastric outlet obstruction	Deposition in the pylorus or proximal duodenum, muscular rigidity, and neuromuscular involvement	Gastric cancer, pyloric obstruction, diabetic gastroparesis, or adverse drug effects	Deep deposition should remain a concern despite minor surface abnormalities on endoscopy
Tumor-like phenotype	Weight loss, epigastric pain, bleeding, or obstruction	Nodules, polyps, masses, phytobezoar-like lesions, or early cancer-like lesions	Focal deposition in the lamina propria and submucosa	Gastric cancer, MALT lymphoma, or polyps	Amyloid staining should be added when routine histopathology does not explain the lesion
Ulcerative phenotype	Refractory epigastric pain, melena, and anemia	Refractory ulcers, erosions, and surrounding mucosal edema	Mucosal ischemia, vascular deposition, and local tissue fragility	Ordinary peptic ulcer disease or Helicobacter pylori-associated ulcer	A systemic cause should be considered when conventional therapy fails
Occult phenotype	Nonspecific dyspepsia, minor endoscopic abnormalities, or discordance between symptoms and endoscopy	Near-normal findings, mild inflammatory changes, or surface findings disproportionate to symptoms	Deep muscular or neuromuscular deposition with minimal superficial mucosal involvement	Functional dyspepsia or adverse drug effects	Refractory symptoms and a history of plasma-cell disease are more informative than endoscopic appearance

**Figure 5:**
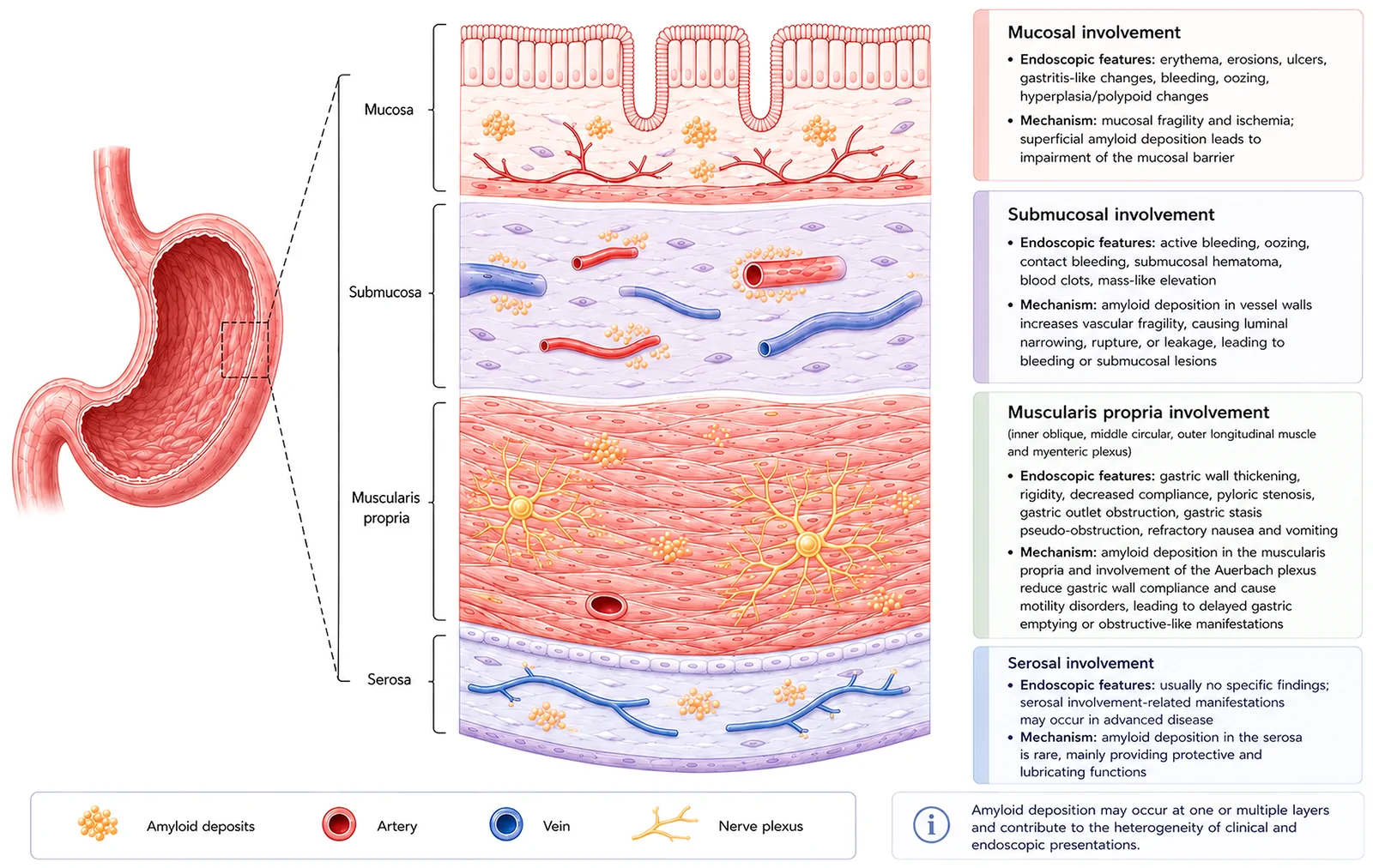
Layer-based clinicopathological model of gastric amyloid deposition.

Although imaging data were limited, they helped explain discordance between endoscopic findings and symptoms. Endoscopy primarily assesses the mucosal surface, whereas amyloid may involve submucosal vessels, the muscularis mucosae, muscularis propria, and neuromuscular structures. Concentric thickening of the antrum and pylorus reported by Inayat et al. (34) was consistent with diffuse mural deposition; gastric dilatation with collapse of the proximal duodenum reported by Cohen et al. (19) reflected gastric outlet obstruction; and the intensely enhancing mass-like lesion reported by Yanase et al. (25) was compatible with an intramural hematoma or vascular lesion. Imaging is therefore valuable for identifying deep mural involvement and the risk of complications rather than for directly establishing gastric amyloidosis. When endoscopic biopsy shows only inflammation, a polyp, or another superficial lesion despite refractory symptoms, or when gastric wall thickening, gastric dilatation, pyloric stenosis, duodenal collapse, or a focally enhancing mass is present, the endoscopic surface findings may underestimate deeper deposition. In such patients, CT, endoscopic ultrasonography, or other imaging may usefully complement endoscopic biopsy.

The nonspecific endoscopic appearance is an important source of misdiagnosis. When gastric lesions occur in a patient with MM, endoscopists and pathologists commonly first consider gastric cancer, MALT lymphoma, peptic ulcer disease, polyps, drug-induced gastritis, or Helicobacter pylori-associated disease. In the index case, endoscopy showed proliferative lesions in the gastric fundus and body, routine histopathology initially identified fundic gland polyps, and a MALT-related lesion was considered endoscopically. This sequence illustrates the ability of gastric amyloidosis to mimic other disorders. The gastric cancer-like case reported by Mathialagan and Sowmya (28), early cancer-like lesion reported by Peng et al. (29), phytobezoar-like mass reported by Marques et al. (27), and refractory gastric ulcer reported by Shih et al. (8) likewise show that no single endoscopic feature is specific for gastric amyloidosis. For hematologists, the more relevant clue is discordance between endoscopic findings and the clinical context, including persistent symptoms, poor response to conventional treatment, an inadequate histopathologic explanation, monoclonal immunoglobulin abnormalities, or relapsed or progressive myeloma.

Histopathologic assessment should not stop at identifying amyloid deposition but should also establish the amyloid protein type. Congo red staining and apple-green birefringence under polarized light are the classic methods of confirmation. In patients with MM or MGUS, however, demonstrating amyloid alone is insufficient for clinical decision-making. Older patients may have both a monoclonal immunoglobulin and non-AL amyloidosis, and immunohistochemistry may be falsely negative or inconclusive because of conformational changes in light-chain fragments. Sidiqi and Gertz (2) identified mass spectrometry-based proteomics as an important method for amyloid typing, particularly when immunohistochemistry is unreliable or the clinical background is complex. In the index case, weakly positive Congo red staining of the gastric tissue, together with relapsed IgAλ MM and improvement in symptoms after anti-multiple myeloma treatment, supported clinical consideration of gastric amyloidosis.

Autopsy and deep-biopsy findings further demonstrate that the pathologic basis of gastric amyloidosis may extend beyond the mucosa. In the autopsy reported by Yamane et al. (37), amyloid deposition extended from the esophagus to the rectum and was particularly prominent in the muscularis mucosae, submucosal vascular walls, and muscularis propria; mural thickening caused stenosis from the pylorus to the postbulbar duodenum. Reithmeier et al. (31) documented involvement of the entire gastrointestinal tract through repeated biopsies extending into the submucosa. These observations explain three common clinical discrepancies. First, some patients have minimal gross endoscopic abnormalities despite severe symptoms because the principal lesions involve the muscular or neuromuscular layers. Second, superficial biopsy may show inflammation, a polyp, or an ulcer while the disease continues to progress because the sample does not adequately represent deeper deposition. Third, refractory vomiting, gastric retention, or pseudo-obstruction may occur without a discrete mechanical tumor because muscular rigidity, neuromuscular involvement, and vascular deposition collectively impair motility or narrow the lumen. These pathologic data therefore explain discordance among endoscopic, imaging, and symptomatic findings.

Management must address both local complications and the abnormal plasma-cell clone. In the bleeding phenotype, acid suppression, transfusion, endoscopic evaluation and hemostasis, interventional radiology, or surgery may be used for acute management. When bleeding originates from extensive amyloid deposition in vascular walls, however, local treatment alone is unlikely to reduce the underlying risk of recurrence. In the obstructive phenotype, gastrointestinal decompression, nutritional support, stenting, or surgery should be selected according to disease extent and overall condition, while etiologic therapy remains centered on control of the abnormal plasma-cell clone. Cohen et al. (19) showed that nonsurgical supportive care combined with plasma-cell-directed treatment could restore oral intake in some patients with gastric outlet obstruction. Bhagat et al. (23) reported regression of a localized gastric amyloid mass after anti-multiple myeloma treatment. In the index patient, nausea and vomiting gradually improved after anti-multiple myeloma treatment was resumed, and repeat endoscopy after two treatment cycles showed fewer and smaller polypoid elevations. Thus, during part of the clinical course, the gastric symptoms and some endoscopic lesions may have improved with control of the plasma-cell clone. As the disease subsequently progressed, nausea and vomiting recurred intermittently and repeat endoscopy showed a gradual increase in the polypoid elevations, consistent with renewed gastric involvement when the underlying disease was inadequately controlled or progressed again. This longitudinal course supports management directed beyond local endoscopic treatment or symptomatic antiemetic therapy. Sustained and effective control of the abnormal plasma-cell clone remains central, together with management of local complications such as bleeding, obstruction, malnutrition, and gastric dysmotility according to the patient’s overall condition.

Clinically overt gastric involvement may not be an isolated event and may reflect a high systemic disease burden. Among the pooled cases, outcomes were reported in 15 patients: 8/15 (53.3%) died; 6/15 (40.0%) had symptom improvement, cessation of bleeding, regression of gastric lesions, or short-term postoperative stability; and 1/15 (6.7%) transitioned to palliative care because anti-multiple myeloma treatment was not tolerated. Menke et al. (4) reported poor outcomes among patients with symptomatic gastric amyloidosis, and the real-world study by Palladini et al. (38) showed that outcomes in AL amyloidosis are closely related to the extent of organ involvement, treatment era, and depth of clonal response. Poor prognosis may be related to diagnostic delay, multiorgan involvement, nutritional deterioration caused by bleeding or obstruction, limitations on treatment intensity imposed by cardiac or renal involvement, and relapsed/refractory myeloma. The index patient relapsed after discontinuing treatment twice and ultimately died of disease progression approximately 1 year after the onset of gastric symptoms. Interruption of maintenance therapy, increased light-chain burden during relapse, and gastric amyloidosis may therefore have jointly reflected an adverse disease course. Once gastric symptoms emerge, hematologic reassessment of myeloma response status, systemic involvement, and treatment tolerance should accompany management of the gastrointestinal disorder.

Overall, MM-associated gastric amyloidosis can be conceptualized as a continuum. At the clonal level, abnormal plasma cells produce potentially amyloidogenic light chains. All heavy-chain types reported in the published cases were IgG, whereas the IgAλ type in the index case shows that the disorder is not confined to IgG myeloma. At the tissue level, amyloid deposits in the mucosa, submucosal vessels, muscularis mucosae, muscularis propria, and neuromuscular structures. Structurally, the gastric wall may become thickened or rigid or develop hematoma, stenosis, mass-like changes, or dilatation. Clinically, patients may present with hematemesis, melena, submucosal hematoma, refractory nausea and vomiting, gastric retention, gastric outlet obstruction, gastric cancer-like lesions, or refractory ulcers. Diagnostically, some patients are found to have myeloma after investigation of a gastric lesion, whereas others develop gastric involvement during relapse or progression of known myeloma. This progression from clonal abnormality to gastric wall deposition, structural change, and clinical phenotype extends the analysis beyond that of isolated case reports.

Based on the index case and the pooled literature, we propose a clinical framework for recognizing MM-associated gastric amyloidosis (**Figure 6**). Gastric amyloidosis should be considered in patients with MM, SMM, MGUS, or another plasma-cell disorder who develop recurrent upper gastrointestinal bleeding, melena, hematemesis, refractory nausea and vomiting, gastric retention, pyloric stenosis, gastric outlet obstruction, a refractory gastric ulcer, a gastric cancer-like lesion, MALT-like proliferative changes, submucosal hematoma, or a gastric lesion unexplained by routine histopathology (2, 3, 39). Endoscopic assessment should include multiple adequate biopsies, with deeper tissue obtained after evaluation of bleeding risk when necessary. Congo red staining should be followed by confirmation under polarized light and κ/λ immunohistochemistry or mass spectrometric typing. Serum and urine immunofixation electrophoresis, serum free light-chain testing, and bone marrow evaluation should be performed concurrently, together with assessment for systemic organ involvement (2). Conversely, when gastric biopsy first identifies amyloid, an underlying plasma-cell disorder should be actively investigated.

**Figure 6:**
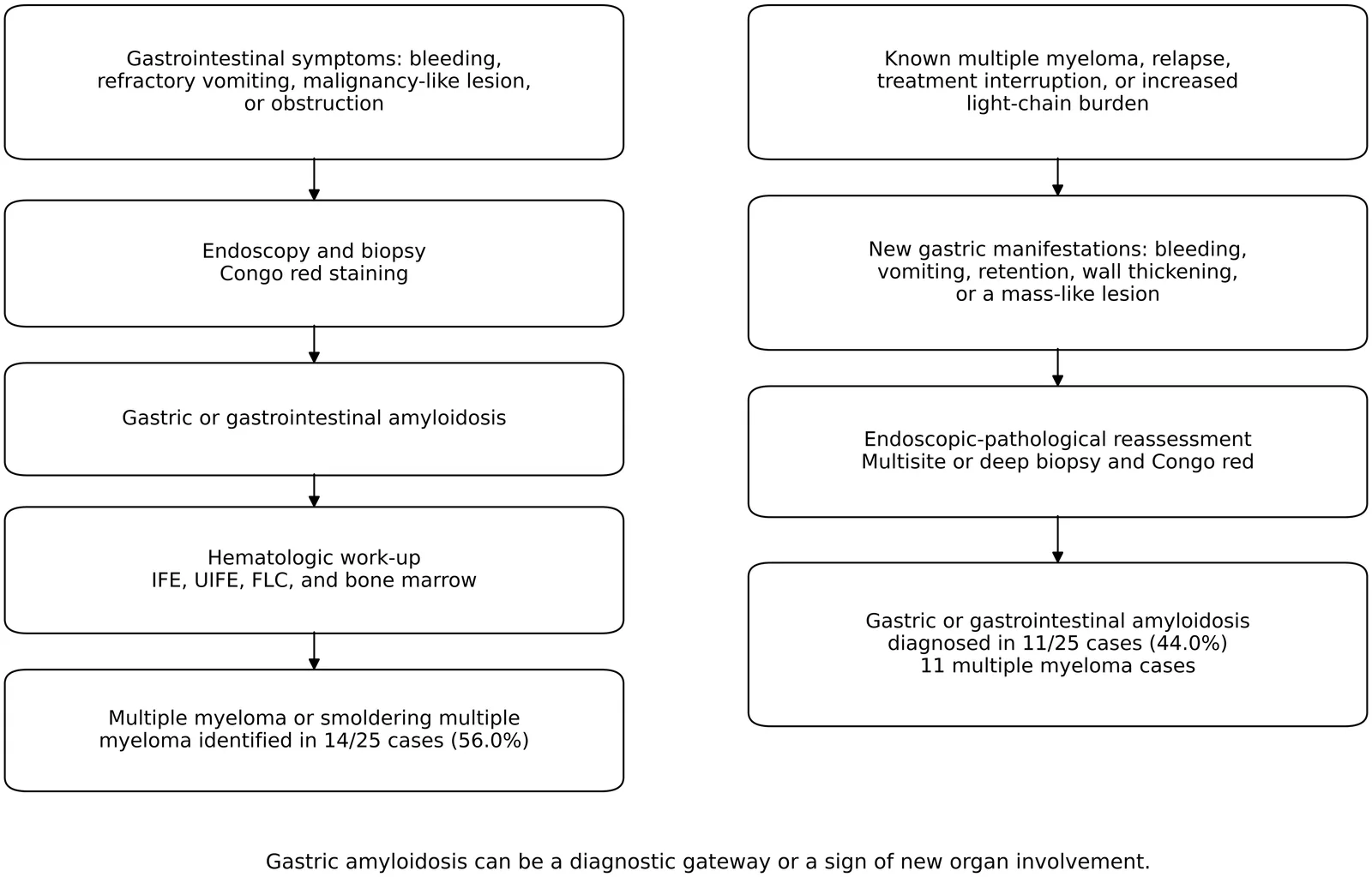
Bidirectional diagnostic pathways between gastric amyloidosis and plasma-cell disorders.

This study has two principal innovations. First, serial endoscopic examinations in April, June, September, and December 2023 provide, to our knowledge, the first longitudinal demonstration of gastric endoscopic lesions changing with response to anti-multiple myeloma treatment and subsequent disease progression. The polypoid elevations became fewer and smaller after treatment and increased again as the disease continued to progress, providing serial endoscopic evidence of a possible dynamic association between gastric manifestations and plasma-cell clonal activity. Second, based on the index case and the pooled analysis of 30 published patients, we propose, to the best of our knowledge for the first time, that gastric amyloidosis may be classified into bleeding, obstructive, tumor-like, ulcerative, and occult clinical-endoscopic phenotypes. Relating these phenotypes to the layer of amyloid deposition and the involved structures provides a framework for interpreting their clinical manifestations, endoscopic or imaging features, and common diagnostic pitfalls and may facilitate clinical recognition and stratified assessment.

This study has several limitations. First, the pathologic evidence in the index case was limited. The gastric mucosal biopsy underwent only routine histopathologic examination and Congo red staining. Because of limitations in the pathology laboratory and the condition of the archived specimen, polarized light microscopy could not be performed to assess characteristic apple-green birefringence, and neither κ/λ immunohistochemistry nor mass spectrometry was available to determine the amyloid protein type. Although the patient had IgA-λ MM with a monoclonal λ light-chain abnormality and her gastric symptoms and endoscopic lesions appeared to vary with myeloma disease activity, supporting clinical consideration of MM-associated AL gastric amyloidosis, the absence of confirmation under polarized light and amyloid protein typing limited standardized histopathologic confirmation and definitive classification. Second, the pooled data were derived mainly from case reports that differed in diagnostic era, biopsy depth, quality of Congo red staining, use of polarized light microscopy, light-chain typing methods, and duration of follow-up. Some lacked complete outcome data, precluding rigorous statistical inference. Publication bias was also likely: severe or unusual manifestations such as bleeding, obstruction, masses, and hematomas are more likely to be reported, whereas occult and mild cases may be underrepresented. Nevertheless, the clinical spectrum derived from 30 patients remains useful. The principal clues to MM-associated gastric amyloidosis are not any single endoscopic appearance, but persistent and refractory symptoms, lesions that mimic other disorders, discordance between clinical and histopathologic findings, and temporal concordance between gastric manifestations and myeloma activity. For hematology and gastrointestinal endoscopy teams, proactively requesting Congo red staining and tissue typing is central to reducing diagnostic delay, avoiding misdiagnosis, and guiding systemic treatment.

## 5 Conclusion

MM-associated gastric amyloidosis is rare and phenotypically diverse. Common clues include gastrointestinal bleeding, refractory nausea and vomiting, gastric outlet obstruction, refractory ulcers, and tumor-like lesions. Endoscopy and superficial biopsy may underestimate deeper gastric wall involvement. In suspected cases, representative tissue should be obtained through multidisciplinary collaboration, followed by Congo red staining, confirmation under polarized light, and amyloid protein typing; plasma-cell clonal activity and involvement of other organs should be assessed concurrently. During treatment, longitudinal changes in gastrointestinal symptoms and endoscopic lesions may provide clinically useful evidence of systemic treatment response and disease progression. A complete diagnostic evidence chain, early recognition, and sustained control of the underlying disease are central to improving clinical management.

## Data Availability

The original contributions presented in the study are included in the article/**Supplementary Material**, further inquiries can be directed to the corresponding authors.
