# Supplementary material for: Gastric involvement in multiple myeloma-associated AL amyloidosis: a case with serial endoscopy and a pooled analysis of 30 published cases
Source: Front Oncol. 2026 Oct 6;16:1960604. doi: 10.3389/fonc.2026.1960604 (PMC13680210; doi:10.3389/fonc.2026.1960604)
Supplement: Supplementary file 1 [file Table1.docx]

**Supplementary Table 1. Statistical information for 30 patients**

| **Author** | **Publication year** | **Sex** | **Age** | **Symptoms** | **Site(s) of involvement** | **Endoscopic findings** | **Imaging findings** | **Treatment** | **Outcome** | **Diagnostic method** | **Diagnostic sequence** | **Diagnosis** | **Immunoglobulin type** | **Light-chain type** |
| --- | --- | --- | --- | --- | --- | --- | --- | --- | --- | --- | --- | --- | --- | --- |
| Gjeorgjievski M [1] | 2015 | Female | 92 | Melena | Gastric body along the lesser curvature | A large hemispherical 11 × 6 cm adherent blood clot overlying the gastric body, with a 2.5 × 2 cm ulcerated mass | NA | Pantoprazole was administered intravenously as an 80 mg loading dose followed by an infusion at 2 mg/h; 2 units of packed red blood cells were transfused, and omeprazole 40 mg was administered orally once daily. No further bleeding occurred during 1 month of follow-up. | Improved | Biopsy and Congo red staining | NA | Smoldering multiple myeloma (SMM) | IgG | λ |
| Singh K [2] | 2016 | Male | 61 | Hematemesis and melena | Gastric antrum, body, and distal stomach | Gastric ulcers and red nodular ulcerations | NA | Acid suppression and gastric mucosal protection | NA | Biopsy | Multiple myeloma diagnosed first | Multiple myeloma | NA | NA |
| Shih IC [3] | 2022 | Female | 69 | Epigastric pain | Gastric antrum | Gastric antral ulcer with diffuse nodular changes (polypoid nodules) | NA | Anti-multiple myeloma treatment with melphalan, thalidomide, and dexamethasone | Improved | Biopsy and Congo red staining | Amyloidosis diagnosed first | Multiple myeloma | NA | κ |
| Chan R [4] | 2021 | Male | 76 | Hematemesis and melena | Gastric fundus, cardia, and antrum | Mucosal erosions and nodular hyperplasia with gastritis in the gastric fundus, cardia, and antrum | NA | NA | NA | Congo red staining; biopsy showed active chronic gastritis with ulceration | NA | Multiple myeloma | NA | NA |
| Liu AL [5] | 2022 | Female | 63 | Abdominal distension, abdominal pain, and hematochezia | Gastric angle | Upper endoscopy showed gastric retention and rough, congested mucosa at the gastric angle with slight oozing. Colonoscopy showed mucosal congestion and edema in the distal ascending and sigmoid colon, multiple round and irregular ulcers, submucosal ecchymoses, and hematoma formation. | NA | Anti-multiple myeloma treatment | Improved | Biopsy and Congo red staining | Amyloidosis diagnosed first | Smoldering multiple myeloma (SMM) | NA | NA |
| Alvares JF [6] | 2003 | Female | 45 | Hematemesis | Gastric antrum | Nodular lesion and ulcer in the gastric antrum | NA | Anti-multiple myeloma treatment | NA | Biopsy and Congo red staining | Multiple myeloma diagnosed first | Multiple myeloma | NA | NA |
| Ayabe T [7] | 1993 | Male | 65 | Bleeding gastric ulcer | Stomach | Gastric ulcer and red nodular ulceration | NA | NA | NA | NA | Amyloidosis diagnosed first | Multiple myeloma | NA | NA |
| Khan Z [8] | 2017 | Female | 67 | Active bleeding | Distal gastric body, proximal antrum, and proximal transverse colon | A large mass along the curvature of the distal gastric body and proximal antrum with active bleeding; a submucosal hematoma surrounded the 3 × 7 cm mass. Colonoscopy showed a large clot-covered mass with fresh bleeding in the proximal transverse colon. | NA | Partial gastrectomy; conservative management of the colonic lesion | No further gastrointestinal bleeding during follow-up | Biopsy demonstrated gastrointestinal amyloidosis | NA | Multiple myeloma, atrial fibrillation, and ongoing anticoagulation with rivaroxaban | NA | NA |
| Zhou HB [9] | 2018 | Female | 63 | Hematemesis | Gastric antrum and ascending colon | Ulcer | NA | Anti-multiple myeloma treatment with cyclophosphamide (Jiangsu Hengrui Pharmaceuticals Co., Ltd., China) 400 mg on days 1–4, dexamethasone (Xianju Pharmaceutical Co., Ltd.) 20 mg on days 1–4 and 15–18, and thalidomide (Changzhou Pharmaceutical Co., Ltd.) 50 mg nightly | Improved and discharged | Biopsy and Congo red staining | Amyloidosis diagnosed first | Multiple myeloma | IgG | λ |
| Chapple CR [10] | 1986 | Male | 73 | Vomiting | NA | NA | NA | Anti-multiple myeloma treatment | NA | Biopsy | Amyloidosis diagnosed first | Multiple myeloma | IgG | NA |
| Cohen JA [11] | 2016 | Male | 64 | Nausea and vomiting | Gastric antrum | Polypoid lesion in the gastric antrum with pyloric edema | Abdominal CT showed marked gastric distension with collapse of the proximal duodenum. | Carfilzomib, cyclophosphamide, and dexamethasone | NA | Biopsy and Congo red staining | Multiple myeloma diagnosed first | Multiple myeloma | NA | NA |
| Lei GY [12] | 2020 | Female | 59 | Vomiting | Pylorus | Inflamed, stenotic pylorus with contact bleeding | NA | Anti-multiple myeloma treatment | Death | Biopsy | Multiple myeloma diagnosed first | Multiple myeloma | NA | NA |
| Liu W [13] | 2011 | Female | 62 | Abdominal distension, poor appetite, and vomiting | NA | Upper endoscopy suggested (1) gastric retention and (2) gastric cancer. | NA | NA | NA | Biopsy and Congo red staining | NA | Multiple myeloma | NA | NA |
| Wald A [14] | 1981 | Female | 39 | Nausea, vomiting, abdominal distension, abdominal pain, and upper gastrointestinal bleeding | Gastrointestinal tract | NA | NA | NA | Death | NA | Multiple myeloma diagnosed first | Multiple myeloma | NA | NA |
| Bhagat G [15] | 2009 | Male | 82 | Vomiting and hematemesis | Pylorus | Annular mass at the gastric outlet causing pyloric obstruction | NA | Anti-multiple myeloma treatment | Complete regression of the mass after 6 months of anti-multiple myeloma treatment | Biopsy | Multiple myeloma diagnosed first | Multiple myeloma | NA | κ |
| Kim SY [16] | 2016 | Male | 71 | Dyspepsia and epigastric burning | Gastric fundus and body | Initial endoscopy revealed a very large submucosal hematoma on the anterior wall extending from the fundus to the upper gastric body. Follow-up endoscopy showed an extensive submucosal hematoma involving the entire upper half of the stomach. The hematoma continued to expand. | NA | Total gastrectomy followed by postoperative anti-multiple myeloma treatment | NA | Biopsy and Congo red staining | Amyloidosis diagnosed first | Multiple myeloma | NA | λ |
| Yanase Y [17] | 2015 | Female | 43 | Hematemesis | Gastric antrum | A giant submucosal hematoma in the gastric antrum with contact bleeding and a giant ulcer; multiple submucosal hematomas and ulcers | Abdominal CT showed a markedly enhancing mass with heterogeneous internal density in the gastric antrum | Anti-multiple myeloma treatment | NA | Biopsy and Congo red staining | Amyloidosis diagnosed first | Multiple myeloma | IgG | κ |
| Pak SC [18] | 2017 | Female | 67 | Abdominal pain and hematemesis | Greater curvature of the stomach | Polypoid mass and hematoma | NA | NA | NA | Biopsy, Congo red staining, and sulfated Alcian blue staining | Multiple myeloma diagnosed first | Multiple myeloma | NA | NA |
| Marques M [19] | 2011 | Male | 55 | Hematemesis | Gastric antrum and body | A large vegetating mass covered with white plaques at the junction of the gastric body and antrum; white plaque infiltration of the gastric mucosa with mucosal oozing | NA | Surgery | Death | Biopsy | Multiple myeloma diagnosed first | Multiple myeloma | NA | NA |
| Mathialagan J [20] | 2020 | Male | 55 | Dysphagia | Gastric antrum and duodenum | Gastritis and duodenitis | NA | NA | NA | Biopsy | Amyloidosis diagnosed first | Multiple myeloma | NA | NA |
| Peng J [21] | 2025 | Female | 65 | Abdominal distension and abdominal pain | Junction of the gastric antrum and body | A 0-IIa+IIc lesion measuring approximately 3 × 6 cm was identified at the junction of the gastric antrum and body; the mucosal surface was smooth and bled easily on contact | NA | Anti-multiple myeloma treatment | NA | Biopsy and Congo red staining | Amyloidosis diagnosed first | Multiple myeloma | NA | NA |
| Shirahama M [22] | 1991 | Male | 70 | Alternating diarrhea and constipation, with hematochezia | NA | Endoscopy showed no obvious gastric mucosal abnormalities. | NA | NA | Death | Biopsy | Multiple myeloma diagnosed first | Multiple myeloma | NA | NA |
| Reithmeier A [23] | 1995 | Male | 50 | Dyspepsia, abdominal pain, and abdominal distension | NA | Upper endoscopy showed grossly normal mucosa from the stomach to the ligament of Treitz; histologic examination was also normal. | NA | Anti-multiple myeloma treatment | Death | Biopsy | Multiple myeloma diagnosed first | Multiple myeloma | IgG | κ |
| Pelletier AL [24] | 2007 | Male | 50 | Hematemesis | Gastric antrum and lesser curvature | A 3-cm granulomatous lesion in the gastric antrum and along the lesser curvature, with diffuse hemorrhagic mucosal oozing | Prior anticoagulant therapy for angina pectoris | Transplantation | NA | Biopsy | Amyloidosis diagnosed first | Multiple myeloma | NA | NA |
| Xiao H [25] | 2020 | Female | 68 | Loss of appetite, vomiting, and melena | Esophagus and gastric antrum | First examination: congested, swollen, rough, and eroded mucosa in the middle and lower esophagus; nodular mucosal elevations with antral erosions, a rigid gastric wall, and pyloric stenosis. Second examination: congested and swollen mucosa in the gastric fundus, body, angle, and antrum, with an elevated antral mucosal lesion showing surface erosion and findings suggestive of gastric cancer. | NA | The patient died of multiorgan failure before anti-multiple myeloma treatment could be initiated after diagnosis | Death | Biopsy and Congo red staining | NA | Multiple myeloma | IgG | λ |
| Inayat F [26] | 2019 | Female | 56 | Epigastric pain, nausea, and vomiting | Gastric antrum, pylorus, and duodenum | Diffuse nodular changes in the gastric antrum, pylorus, and descending duodenum | Contrast-enhanced abdominal CT showed concentric thickening of the gastric antrum and pylorus | Cyclophosphamide, bortezomib, and dexamethasone (CyBorD) | NA | Biopsy and Congo red staining | Amyloidosis diagnosed first | Multiple myeloma, cardiac amyloidosis, heart failure, and atrial flutter | NA | κ |
| McFarlane M [27] | 2018 | Male | 59 | Vomiting and constipation | Stomach | Gastritis and bleeding | NA | Anti-multiple myeloma treatment | Unable to tolerate anti-multiple myeloma treatment; palliative care | Biopsy and Congo red staining | Amyloidosis diagnosed first | Multiple myeloma | NA | κ |
| Ghazaleh S [28] | 2021 | Male | 81 | Dysphagia | Gastric antrum | Patchy erythema in the gastric antrum | NA | Anti-multiple myeloma treatment | NA | Biopsy and Congo red staining | Amyloidosis diagnosed first | Multiple myeloma | NA | κ |
| Yamane T [29] | 2005 | Male | 54 | Nausea and vomiting | Pylorus | Distal pyloric stenosis | NA | NA | Death | Biopsy | Multiple myeloma | Multiple myeloma | NA | NA |
| Suchartlikitwong S [30] | 2018 | Female | 59 | Hematochezia | Gastric antrum | Congested and erythematous gastric mucosa with a single 8 mm sessile polyp in the gastric antrum. | NA | Anti-multiple myeloma treatment | Death | Biopsy and Congo red staining | Amyloidosis diagnosed first | Multiple myeloma | NA | κ |

**References**

[1] Gjeorgjievski M, Purohit T, Amin MB, Kurtin PJ, Cappell MS. Upper gastrointestinal bleeding from gastric amyloidosis in a patient with smoldering multiple myeloma. Case Rep Gastrointest Med. 2015;2015:320120. doi: 10.1155/2015/320120. PMID: 26366309; PMCID: PMC4561092.

[2] Singh K, Chapalamadugu P, Malet P. Upper gastrointestinal bleeding due to amyloidosis in a patient with multiple myeloma. Clin Gastroenterol Hepatol. 2016;14(11): A22-A23. doi: 10.1016/j.cgh.2016.07.015. PMID: 27451090.

[3] Shih IC, Chen HC, Chuang CH. An unusual cause of refractory gastric ulcer. Gastroenterology. 2023;164(7):1059-1061. doi: 10.1053/j.gastro.2022.12.021. PMID: 36592734.

[4] Chan R, Carpentier S. Gastric amyloidosis presenting as acute upper gastrointestinal bleeding: a case report. BMC Gastroenterol. 2021;21(1):300. doi: 10.1186/s12876-021-01882-7. PMID: 34325671; PMCID: PMC8323269.

[5] Liu AL, Ding XL, Liu H, Zhao WJ, Jing X, Zhou X, et al. Gastrointestinal amyloidosis in a patient with smoldering multiple myeloma: a case report. World J Clin Cases. 2022;10(7):2307-2314. doi: 10.12998/wjcc.v10.i7.2307. PMID: 35321167; PMCID: PMC8895179.

[6] Alvares JF, Thomas J, Ramakrishna K, Rao L, Rao AC. Multiple myeloma with amyloidosis presenting with recurrent gastrointestinal bleeding. Indian J Gastroenterol. 2003;22(5):196. PMID: 14658543.

[7] Ayabe T, Maekawa I, Ashida T, Taruishi M, Shibata Y, Namiki M. [A case of gastric amyloidosis developing into Bence-Jones type multiple myeloma, treated effectively by cyclo-VAMP chemotherapy]. Gan To Kagaku Ryoho. 1993;20(15):2399-403. Japanese. PMID: 8259860.

[8] Khan Z, Darr U, Renno A, Tiwari A, Sofi A, Nawras A. Massive upper and lower gastrointestinal bleeding from simultaneous primary (AL) amyloidosis of the stomach and transverse colon in a patient with multiple myeloma. Case Rep Gastroenterol. 2017;11(3):625-631. doi: 10.1159/000480073. PMID: 29282382; PMCID: PMC5731163.

[9] Zhou HB. A case of multiple myeloma initially presenting as hematemesis. Turk J Gastroenterol. 2018;29(1):108-111. doi: 10.5152/tjg.2018.17710. PMID: 29391314; PMCID: PMC6322629.

[10] Chapple CR, Chesner IM, Newman J. Multiple myeloma presenting as intractable gastric retention in a patient with a previous gastroenterostomy. Br J Surg. 1986;73(11):930. doi: 10.1002/bjs.1800731132.

[11] Cohen JA, An J, Brown AW, Spearman D, Paredes A. Gastric outlet obstruction due to gastrointestinal amyloidosis. J Gastrointest Surg. 2017;21(3):600-601. doi: 10.1007/s11605-016-3248-2. PMID: 27542374.

[12] Lei GY, Bundele MM, Shelat VG. Gastric outlet obstruction from gastric amyloidosis caused by multiple myeloma. Clin Gastroenterol Hepatol. 2021;19(6): A21-A22. doi: 10.1016/j.cgh.2020.04.012. PMID: 32289540; PMCID: PMC7770489.

[13] Liu W, Zhou X. Multiple myeloma associated with light-chain amyloidosis manifesting as gastric retention: a case report and review of the literature. West Indian Med J. 2011;60(6):681-4. PMID: 22512230.

[14] Wald A, Kichler J, Mendelow H. Amyloidosis and chronic intestinal pseudoobstruction. Dig Dis Sci. 1981;26(5):462-5. doi: 10.1007/BF01313592. PMID: 7249887.

[15] Bhagat G, Naiyer AJ, Green PH. Regression of a localized gastric amyloid mass in a patient treated for multiple myeloma. Gastrointest Endosc. 2009;69(4):950-1; discussion 951. doi: 10.1016/j.gie.2008.10.050. PMID: 19327484.

[16] Kim SY, Moon SB, Lee SK, Hong SK, Kim YH, Chae GB, et al. Light-chain amyloidosis presenting with rapidly progressive submucosal hemorrhage of the stomach. Asian J Surg. 2016;39(2):113-5. doi: 10.1016/j.asjsur.2013.09.013. PMID: 24246158.

[17] Yanase Y, Waseda Y, Saito N, Oishi G, Okada K, Kobayashi T, et al. [A case of AL amyloidosis due to multiple myeloma presenting with a huge gastric submucosal hematoma during anticoagulant therapy]. Nihon Shokakibyo Gakkai Zasshi. 2015;112(6):1023-9. Japanese. doi: 10.11405/nisshoshi.112.1023. PMID: 26050725.

[18] Pak SC, Markovic JT. Gastric submucosal haematoma caused by an amyloidoma in the setting of multiple myeloma. Arab J Gastroenterol. 2017;18(4):238-240. doi: 10.1016/j.ajg.2017.12.002. PMID: 29249671.

[19] Marques M, Sarmento JA, Rodrigues S, Guimarães S, Fonseca E, Macedo G. Gastric amyloidosis: unusual cause of upper gastrointestinal hemorrhage. Endoscopy. 2011;43 Suppl 2 UCTN: E100-1. doi: 10.1055/s-0030-1256080. PMID: 21424994.

[20] Mathialagan J, Sowmya S. Amyloidosis masquerading as gastric carcinoma - a case report. Indian J Pathol Microbiol. 2020;63(1):119-121. doi: 10.4103/IJPM.IJPM_258_19. PMID: 32031139.

[21] Peng J, Zhang C, Tang X. Gastric amyloidosis as an initial presentation of multiple myeloma. Rev Gastroenterol Mex (Engl Ed). 2025;90(4):594-595. doi: 10.1016/j.rgmxen.2025.10.012. PMID: 41176474.

[22] Shirahama M, Morita S, Koga T, Gotoh Y, Uchida S, Ohta Y, et al. Gastrointestinal amyloidosis associated with multiple myeloma: sonographic features. J Clin Ultrasound. 1991;19(8):493-7. doi: 10.1002/jcu.1870190807. PMID: 1658080.

[23] Reithmeier A, Trenkwalder P, Kaess H, Lydtin H. Gastrointestinale Amyloidose bei IgG-xi-Leichtketten-Plasmozytom. Diagnostische Probleme trotz fortgeschrittenen Befalls [Gastrointestinal amyloidosis in IgG-chi light-chain plasmacytoma: diagnostic problems despite advanced involvement]. Dtsch Med Wochenschr. 1995;120(18):641-5. German.
doi: 10.1055/s-2008-1055390. PMID: 7750431.

[24] Pelletier AL, O'Toole D, Couvelard A, Hentic O, Nahon-Uzan K, Corcos O, et al. Amylose systémique AL révélée par une pseudo-tumeur gastrique hémorragique [Systemic AL amyloidosis revealed by a hemorrhagic gastric pseudotumor]. Gastroenterol Clin Biol. 2007;31(3):310-2. French. doi: 10.1016/s0399-8320(07)89382-1. PMID: 17396094.

[25] Xiao H, Qing D, Li C, Zhou H. A case report of gastric amyloidosis due to multiple myeloma mimicking gastric cancer. BMC Gastroenterol. 2020;20(1):219. doi: 10.1186/s12876-020-01359-z. PMID: 32652937; PMCID: PMC7353757.

[26] Inayat F, Ur Rahman A, Zahid E, Ali NS, Charles R. Symptomatic involvement of the stomach and duodenum as initial presentation of AL amyloidosis. BMJ Case Rep. 2019;12(1): bcr-2018-227550. doi: 10.1136/bcr-2018-227550. PMID: 30659008; PMCID: PMC6340590.

[27] McFarlane M, Bashford A, Sah S, Disney BR. Multisystem amyloidosis as the unifying diagnosis for constipation, collapse and cardiomyopathy. BMJ Case Rep. 2018;2018: bcr2018225301. doi: 10.1136/bcr-2018-225301. PMID: 29991549; PMCID: PMC6047702.

[28] Ghazaleh S, Hindupur A, Nehme C, Khader Y, Ghanim M, Sheikh T, et al. Gastric amyloidosis in a patient with dysphagia. Gastroenterol Hepatol Bed Bench. 2021;14(2):178-180. PMID: 33968346; PMCID: PMC8101516.

[29] Yamane T, Nakamura M, Uchiyama K, Ishii T, Sakurai T, Matsunaga K, et al. [A case of secondary amyloidosis from multiple myeloma with stenosis of the upper digestive tract]. Nihon Shokakibyo Gakkai Zasshi. 2005;102(9):1176-82. Japanese. PMID: 16180676.

[30] Suchartlikitwong S, Tantrachoti P, Mingbunjerdsuk T, Laoveeravat P, Rakvit A. Gastrointestinal polyps and hemorrhage as a presentation of primary systemic light-chain amyloidosis. ACG Case Rep J. 2018;5: e44. doi: 10.14309/crj.2018.44. PMID: 29915792; PMCID: PMC5992335.
